# A 24 month longitudinal cohort study of hookworm infection among school-age children in Ghana: Predictors of persistent and repeated infection

**DOI:** 10.1371/journal.pntd.0014492

**Published:** 2026-07-31

**Authors:** Debbie L. Humphries, Irene A. Larbi, Molly McLaughlin, Yue Hu, Luis Maldonado, Joseph Otchere, Josephine Quagraine, Hibbah Araba Osei-Kwasi, Sena Seddoh, George E. Mensah, Dickson Osabutey, Lisa M. Harrison, Hector Nobiya L. Samani, Kwadwo Ansah Koram, Michael D. Wilson, Michael Cappello

**Affiliations:** 1 Department of Chronic Disease Epidemiology, Yale School of Public Health, New Haven, Connecticut, United States of America; 2 Epidemiology Department, Noguchi Memorial Institute for Medical Research, College of Health Sciences, University of Ghana, Accra, Ghana; 3 Yale College, Yale University, New Haven, Connecticut, United States of America; 4 Parasitology Department, Noguchi Memorial Institute for Medical Research, College of Health Sciences, University of Ghana, Accra, Ghana; 5 Loughborough University, Loughborough, United Kingdom; 6 Epidemiology of Microbial Diseases Department, Yale School of Public Health, New Haven, Connecticut, United States of America; University of Liverpool, UNITED KINGDOM OF GREAT BRITAIN AND NORTHERN IRELAND

## Abstract

**Background:**

Worldwide, 451 million people and up to 156 million children are infected with hookworm, causing an estimated 3.2 million disability-adjusted life-years annually. We conducted a two-year longitudinal cohort study of hookworm infection with a random selection of 274 school-age children (4–16 yrs) in rural Ghana to identify nutritional and environmental exposures impacting hookworm infection and response to treatment.

**Methods:**

Every six months (baseline Jan 2013) we collected anthropometric measurements, pre- and post-treatment fecal samples, and blood samples. Household surveys and multiple-pass twenty-four-hour recalls were conducted at baseline and 18 months.

**Results:**

Seventy-eight participants (28.5%) were absent from at least 1 time point. Of the 196 that were screened at all five time points, 87 (44.4%) were hookworm-infected>1 time. Assessment of albendazole treatment response was conducted on 83 participants. Sixty (72.3%) were cleared of hookworm infections while 23 (27.5%) remained infected post-treatment (ERR: 0–99%). Treatment efficacy was more likely among children aged over 9.66 years (HR: 1.55; 95% CI: 1.18, 2.04) and those from food-insecure households (HR: 2.26; 95% CI: 1.06, 4.81). Hookworm infection status and albendazole treatment outcome at baseline and 18 months were the dominant influences on infections at 6 and 24 months, respectively. Future risk of infection at both 6 and 24 months had significant environmental and nutritional predictors, although the variables differed.

**Conclusions:**

Global school-based deworming has been associated with reduced rates of hookworm infection among schoolchildren in Africa; however, additional strategies will be necessary for long-term, sustainable control. This study reports on an innovative two-year longitudinal study of school age children with repeated cycles of testing and treatment. We modelled hookworm infection as a function of predictors that were measured six months prior, thereby strengthening the assessment of causal relationships between environmental, infection and nutritional risk factors.

## Author summary

Worldwide, 451 million people are estimated to be infected with hookworm, causing an estimated 1.7 million years lived with disability annually [1]. Nutritional status and environmental exposures have been associated with hookworm infections. We conducted a two-year longitudinal cohort study with a random selection of school age children (4–16 yrs) in rural Ghana (n = 274). Every six months (baseline Jan 2013) we collected anthropometric measurements, fecal samples and venous blood samples. Household surveys and dietary intake were assessed at baseline and 18 months. More than half (55.6%/n = 109/196) of participants were hookworm negative every time and 1.1% (n = 3) were infected on all five occasions. Older children were more likely to be cured by treatment with single dose albendazole, as were children from food insecure households. The strongest predictor of infection at six months and 24 months was infection and response to treatment at the previous timepoint. While school-based deworming has been associated with reduced rates of hookworm infection among children in Africa, additional strategies will be needed for long-term sustainable control. This study reports on a two-year longitudinal follow up of schoolchildren featuring repeated cycles of testing and treatment for hookworm infection.

## Introduction

As many as 451 million people and 156 million children in tropical regions are at risk of hookworm infection [2–4], a soil-transmitted helminth (STH) endemic in many resource limited communities worldwide. Hookworm infection significantly impacts human health by causing intestinal blood loss and related complications [5,6], and is responsible for an estimated 3.2 million disability-adjusted life-years annually, resulting in over USD100 billion in global economic losses [2]. Most common in resource-poor areas with limited sanitation and sewage treatment, infection is rarely fatal. However, hookworm infections contribute to chronic undernutrition and increased susceptibility to other infectious diseases [7], both of which are prevalent in regions with high rates of infection, thereby compounding the negative effects of hookworm [8,9].

School-age children in malnutrition-endemic regions are quite susceptible to hookworm infection [10]. Children can endure high-intensity hookworm infections that increase anemia risk and cause detrimental effects on macro- and micronutrient status, growth, and cognition, further compounding the negative effects of malnutrition [11–13]. Epidemiological studies have found that school-age children infected with hookworm have significantly lower height-for-age z scores (HAZ), weight-for-age z scores (WAZ), and Body Mass Index (BMI) for age z scores (BAZ) scores compared to uninfected children from the same region [14–18]. Other adverse effects associated with hookworm infection in children include iron-deficiency anemia due to blood feeding in the gut, as well as reduced birth weight and increased risk of infant mortality among pregnant women infected with hookworm [2,19].

Sub-Saharan Africa has particularly high rates of both hookworm infection [3] and malnutrition [20]. As of 2022, the national prevalence of stunting and wasting in children in Ghana under 5 years was 18% and 6.0%, respectively [21]. According to Ghanaian data from 2016, 32.5% of boys and 18.5% of girls ages 5–19 were underweight [22]. Another study in two neighboring communities in Greater Accra from 2017 studying nutrient intake and nutritional status of schoolchildren aged 6–12 found that 67% of children were stunted, underweight, or anemic [23]. A study conducted in the Kintampo North Municipality region of Ghana in 2011 found that 59% of school-age children from five contiguous communities were infected with hookworm, although there are regional variations in hookworm infection prevalence in these communities [24].

Although different factors contribute to hookworm susceptibility and re-infection, poor nutritional status has been found to increase susceptibility to helminth infections in animal models [25]. Studies of hookworm infection in mice, rats and hamsters using *H. polygryus*, *N. brasiliensis* and *A. ceylanicum* have found deficiencies of zinc, selenium, vitamin A, protein, iron, vitamin E and energy can result in increased adult worm burden, fecundity, and fecal egg counts [10,26–35]. In humans, cross-sectional studies have demonstrated an association between poor nutritional status and intestinal helminth infections, although cross-sectional studies cannot determine directionality of the malnutrition and infection relationship [36,37]. In addition, a small number of epidemiological studies found more severe malnutrition to be associated with more symptoms and severity of hookworm infection [38,39].

There is also evidence that poor nutritional status increases the host’s susceptibility to re-infection with hookworm. In one epidemiological study stunted children were re-infected with hookworm at a higher rate than non-stunted children [40]. According to the results of a meta-analysis of helminth studies and micronutrient status, children who took micronutrient supplements of vitamin A and iron were less likely to get re-infected with hookworm 2–12 months after the intervention compared to children who did not take any supplements [41].

There is limited information on the effect of host nutrition on response to albendazole, one of the few drugs used to treat hookworm infection. However, in a previous study conducted in Ghana that investigated the impact of host nutrition on response to albendazole treatment, an increase in mid-upper arm circumference and blood hemoglobin levels were found to be statistically significant predictors of an improved host response to albendazole, with a reduced probability of remaining infected post-treatment [24]. Since malnutrition and hookworm are co-endemic in many regions, it is important to investigate the impact of host nutrition on de-worming strategies, especially given that mass-drug administration is recommended by the WHO as a primary method to decrease hookworm prevalence in endemic regions [24].

### Objectives

Our objective was to determine how modifiable host factors, including nutritional status and environmental exposure, influence risk of future infection with hookworm, response to albendazole therapy and total number of hookworm infections.

## Methods

### Ethical statement

This study was approved by the Yale University Human Investigations Committee and the Institutional Review Boards of the Noguchi Memorial Institute for Medical Research, the Ghana Health Service, and the Scientific Review Committee and the Institutional Ethics Committee at the Kintampo Health Research Center. Parents or guardians provided written consent for each participant, and each child provided their written or verbal assent depending on ability.

### Study design

This was a two-year longitudinal cohort study of hookworm infection with data collection and laboratory assessment of hookworm status every six months.

### Setting

The Republic of Ghana has endemic infections of the two major human hookworm species, *Ancylostoma duodenale* and *Necator americanus* [42,43]. In January 2013 we launched a two-year longitudinal epidemiological field study enrolling school age children in Kintampo North Municipality of the Bono East Region of Ghana, where the predominant species of hookworm infecting humans is *N. americanus* [24,44–46].

### Subjects

Inclusion criteria required study participants to be 4–16 years of age and residing within the Kintampo North District. A sample size of 240 children was calculated to provide 80% power to detect a difference in re-infection of at least 40% in children with low dietary protein, as compared with a 25% re-infection rate in children with high dietary protein based on published data [47].

### Participant recruitment and enrollment

Participants were randomly selected from all households in eight municipalities previously identified as having moderate to high prevalence of hookworm infection [42]. A household census was conducted by Noguchi staff, and children aged 4–16 years were randomly selected from each of the municipalities in proportion to the overall population of children in that age group from each municipality. The study enrolled 274 school age children, aged 4–16, in January 2013.

**Fig 1:**
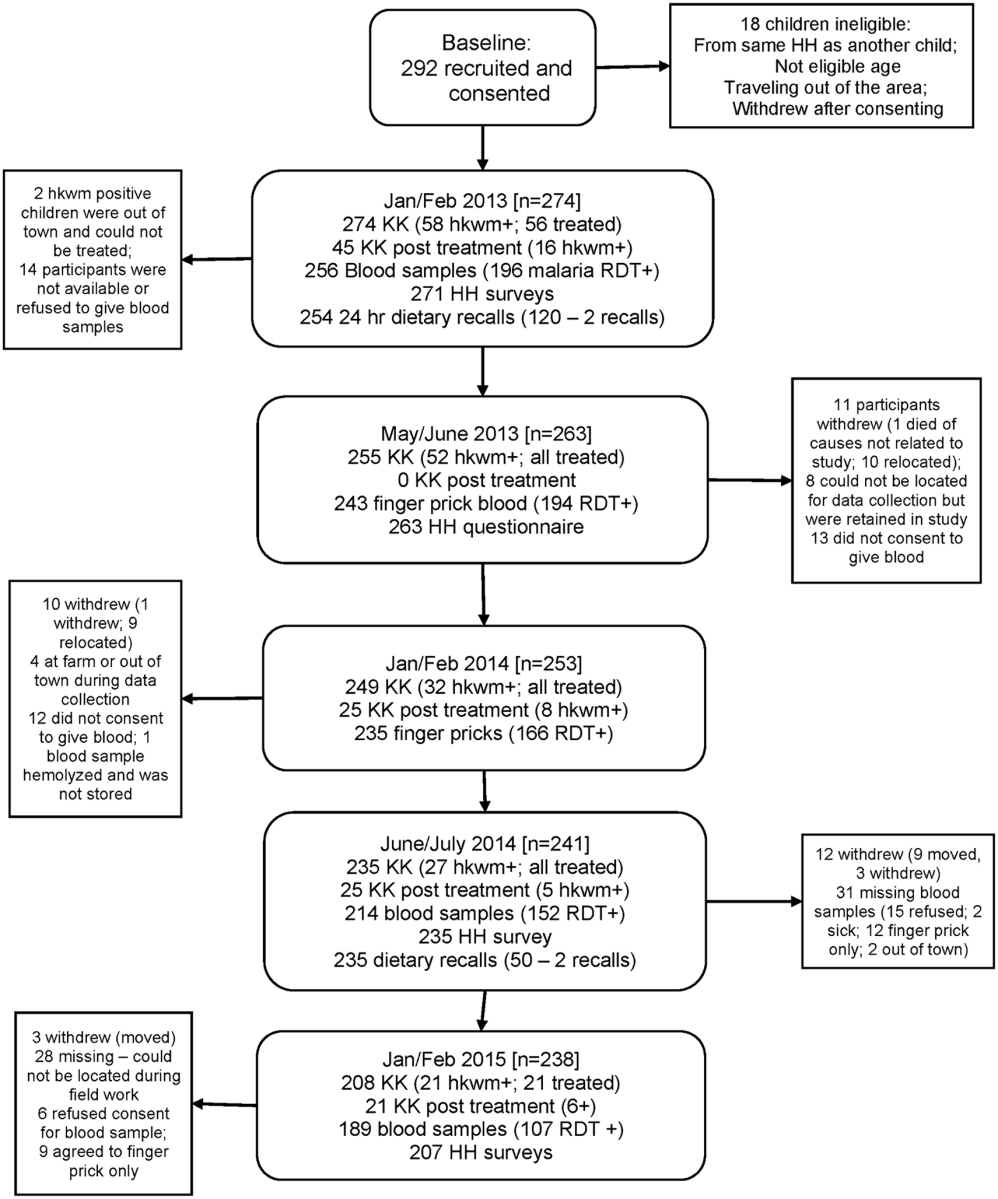
Study Flow diagram. Recruitment, participation and outcomes are shown at each timepoint, as well as disposition of non-participants.

The data collection process was repeated at six-month intervals (baseline: Jan 2013; six months: May-June 2013; 12 months: Jan-Feb 2014; 18 months: June/July 2014; and 24 months: Jan-Feb 2015). At each timepoint anthropometric measurements, fecal and blood samples were collected, and household questionnaires were conducted at baseline and 18 months. Household members provided fecal samples on a rolling basis, with different households sampled at each of the time points to provide an indicator of the child’s exposure at the household level. An overview of the study data collection is provided in Fig 1.

### Laboratory methods

#### Blood samples.

Venous or finger prick blood samples were collected at each timepoint to measure hemoglobin levels and determine the presence and intensity of malaria infection based on thick and thin blood smears [48]. Rapid diagnostic tests (First Response (India) and CareStart (U.S.)) donated by the Ghana Health Service Neglected Tropical Disease Control project were also utilized.

#### Hookworm infection assessment.

At each timepoint stool cups were distributed to the children and/or their guardians and participants were asked to provide a fecal sample. Samples were collected each morning, placed in a cooler for preservation, and driven to the laboratory for processing and analysis. The Kato-Katz technique and microscopy were used in processing and identifying hookworm eggs, as outlined by the WHO [49]. Slides were reviewed within 30 minutes of preparation to ensure accuracy. Eggs per gram were calculated as the sum of eggs counted on two different Kato-Katz slide preps*12. Hookworm-infected participants were referred to local health staff for directly observed oral treatment (albendazole 400 mg). Egg reduction rate was calculated at each time point as 100*(1-epg post treatment/epg pre treatment). Cure rate was calculated as (number of infected participants who were negative following treatment/number of infected participants prior to treatment)x100. Up to five household members from each participant’s household were asked to provide fecal samples at one timepoint for the assessment of household hookworm burden. We report prevalence and intensity of infection at each time point to align our metrics with those utilized by WHO to assess need for hookworm control efforts.

### Household questionnaire

The survey incorporated questions from the Demographic and Health Surveys, the Household Dietary Diversity Score, and the Household Food Insecurity Access Scale (HFIAS) [50,51], and was adapted from a previous survey [42]. All survey materials were translated into the local language (Twi) and back-translated into English by native speakers to ensure accuracy. Questions included birth date, household socioeconomic characteristics, water and sanitation access, access to health care and vaccinations, bed net use, food insecurity, and dietary diversity. Trained local interviewers administered the surveys in the local language. Household assets were used to construct an asset index [52] based on the presence or absence of the following: a tile floor, use of advanced cooking fuel (not charcoal, straw or wood), electricity, radio, TV, phone, refrigerator, bike, car or motorcycle, land, cow, horse or donkey, goat or sheep, pig, poultry, bank or savings account, improved water source, improved toilet). The asset index was used as a continuous variable. Data on parental occupation and education was collected again at 24 months.

### Dietary intake

Multiple pass twenty four hour recalls were collected by trained interviewers at baseline and 18 months [53]. At each timepoint 25% of participants were randomly selected for a repeated twenty four hour recall to estimate intraindividual variation. Estimates of food quantities were developed by a trained nutritionist, based on items purchased and standardized from the local market. Food items were linked with comparable items in the Ghanaian Food Composition Table (University of Ghana), supplemented with items in the Ugandan Food Composition Table where necessary [54], to determine their nutrient contents. Analysis of dietary data used the probability of nutrient adequacy (PNA) [55] approach to estimate adequacy of iron intake, protein intake, and other micronutrients based on the FAO/WHO recommended nutrient intakes when available, and the United States Food and Nutrition Board dietary reference intakes when otherwise [56–60]. Probability of adequacy for nine micronutrients (PNA9; niacin, riboflavin, folate, thiamin, vitamins B_6_ and C, iron, calcium and zinc) were averaged to generate a mean adequacy ratio, and probability of iron adequacy (PNA Iron) was also analyzed separately.

### Determination of anthropometric status

Participant birth dates were confirmed with a household member as part of the household survey, and follow-up visits with a local calendar were used to identify season and year when necessary. Height was measured using a stadiometer, and weight was the average of two digital scale measurements for each child. Anthro Plus [61] was used to calculate z scores based on the World Health Organization growth standards for school age children [62].

### Statistical analysis

#### Total number of infections.

The 274 study participants were assessed for hookworm infections every six months across five time-points. Total number of infections was computed as the sum of a child’s hookworm infections across all timepoints (Table 1a). As the maximum number of expected events per child was 1 for each time-point, our model for total hookworm infections was specified as a linear negative binomial model (NB1), with the log-transformed number of times each participant presented for screening (Table 1b), as an offset. The dispersion statistic for the model, 1.06, suggested an adequate fit to the data [63].

**Table 1:** (a) Frequencies of Numbers of Infection. (b) Frequencies of Participation.

**(a)**			
**Total Number of Infection**	**Frequency**	**Percentage**	**Average Number of Observed Times**
0	166	60.58	4.36 (1.13)
1	63	22.99	4.49 (1.03)
2	20	7.30	4.70 (0.80)
3	15	5.47	4.87 (0.35)
4	7	2.55	5.00 (0.00)
5	3	1.09	5.00 (0.00)
**Total**	**274**	**100**	
**(b)**			
**Number of Times Present**	**Frequency**	**Percentage**	
1	11	4.01	
2	13	4.74	
3	10	3.65	
4	44	16.06	
5	196	71.53	
**Total**	**274**	**100**	

The last two levels of Total Number of Infection were merged in the analysis

Ten percent of the data on the key predictor variables were missing. In making assumptions about the missing data mechanism, we compared cases with and without missing data on the predictor variables. The absence of statistically significant differences favoured the assumption that data were missing at random (Table 2). Predictor variables that were missing ≥30% data were excluded from the analysis. We performed 24 multiple imputations of the data, and assessed convergence by examining trace plots. A complete case analysis, coupled with stepwise model selection, was performed on each of the imputed datasets. Our final pooled model was adjusted for predictor variables selected at least fifty percent of the time in the imputed models [63].

**Table 2:** Hookworm Infections by Participant Socioeconomic Characteristics.

Key Variables at Study Enrollment	TOTAL	HOOKWORM INFECTION ACROSS TIME			
		Never Infected (n = 166)	Infected Once (n = 63)	Infected Twice or More (n = 45)	p-value
**Independent variables/predictors**					
***Individual Variables***					
Age (mean (SD))	9.87 (2.12)	9.80 (2.00)	10.09 (2.24)	9.80 (2.40)	0.633
Gender (n (%))					
Female	137 (50.00)	91 (54.82)	28 (44.44)	18 (40.00)	0.124
Male	137 (50.00)	75 (45.18)	35 (55.56)	27 (60.00)	
BMI-For-Age (mean (SD))	-0.49 (0.87)	-0.55 (0.86)	-0.46 (0.88)	-0.33 (0.93)	0.358
*Observed Data (n (%))*	249 (90.88)	149 (89.76)	60 (95.24)	40 (88.89)	0.381
*Missing Data (n (%))*	25 (9.12)	17 (10.24)	3 (4.76)	5 (11.11)	
Height-For-Age (mean (SD))	-1.13 (1.31)	-1.15 (1.20)	-1.20 (1.37)	-0.92 (1.60)	0.550
*Observed Data (n (%))*	248 (90.51)	148 (89.16)	60 (95.24)	40 (88.89)	0.361
*Missing Data (n (%))*	26 (9.49)	18 (10.84)	3 (4.76)	5 (11.11)	
Ownership of Footwear (n (%))					
No	20 (7.30)	7 (4.22)	8 (12.70)	5 (11.11)	0.037
Yes	250 (91.24)	157 (94.58)	54 (85.71)	39 (86.67)	
*Observed Data (n (%))*	270 (98.54)	164 (98.80)	62 (98.41)	44 (97.78)	0.795
*Missing Data (n (%))*	4 (1.46)	2 (1.20)	1 (1.59)	1 (2.22)	
Frequency of Wearing Footwear (n (%))					
Almost Always	88 (32.12)	56 (33.73)	21 (33.33)	11 (24.44)	0.538
Rarely	182 (66.42)	108 (65.06)	41 (65.08)	33 (73.33)	
*Observed Data (n (%))*	270 (98.54)	164 (98.80)	62 (98.41)	44 (97.78)	0.795
*Missing Data (n (%))*	4 (1.46)	2 (1.20)	1 (1.59)	1 (2.22)	
Probability of Nutrient Adequacy_PNA9 (mean (SD))	0.68 (0.23)	0.70 (0.23)	0.66 (0.26)	0.67 (0.20)	0.571
*Observed Data (n (%))*	216 (78.83)	126 (75.90)	52 (82.54)	38 (84.44)	0.359
*Missing Data (n (%))*	58 (21.17)	40 (24.10)	11 (17.46)	7 (15.56)	
Probability of Iron Adequacy (mean (SD))	0.59 (0.42)	0.60 (0.43)	0.58 (0.43)	0.58 (0.40)	0.962
*Observed Data (n (%))*	216 (78.83)	126 (75.90)	52 (82.54)	38 (84.44)	0.359
*Missing Data (n (%))*	58 (21.17)	40 (24.10)	11 (17.46)	7 (15.56)	
Blood Haemoglobin Level (mean (SD))	11.49 (1.27)	11.46 (1.25)	11.54 (1.36)	11.52 (1.23)	0.917
*Observed Data (n (%))*	257 (93.80)	154 (92.77)	60 (95.24)	43 (95.56)	0.824
*Missing Data (n (%))*	17 (6.20)	12 (7.23)	3 (4.76)	2 (4.44)	
Average Number of study visits completed (mean (SD))	4.46 (1.05)	4.36 (1.13)	4.49 (1.03)	4.82 (0.58)	0.028
***Infection Characteristics***					
Household Prevalence of Hookworm Infection (pooled results from all time points) (n (%))					
No infection	120 (43.80)	82 (49.40)	21 (33.33)	17 (37.78)	0.060
< 0& < 50% prevalence	74 (27.01)	40 (24.10)	22 (34.92)	12 (26.67)	
51–100% prevalence	49 (17.88)	23 (13.86)	14 (22.22)	12 (26.67)	
*Observed Data (n (%))*	243 (88.69)	145 (87.35)	57 (90.48)	41 (91.11)	0.767
*Missing Data (n (%))*	31 (11.31)	21 (12.65)	6 (9.52)	4 (8.89)	
Household Deworming (n (%))					
Non-Treated	146 (53.28)	79 (47.59)	37 (58.73)	30 (66.67)	0.052
Treated	121 (44.16)	82 (49.40)	25 (39.68)	14 (31.11)	
*Observed Data (n (%))*	267 (97.45)	161 (96.99)	62 (98.41)	44 (97.78)	1.000
*Missing Data (n (%))*	7 (2.55)	5 (3.01)	1 (1.59)	1 (2.22)	
Malaria Infection					
Non-Infected	60 (21.90)	40 (24.10)	13 (20.63)	7 (15.56)	0.376
Infected	196 (71.53)	113 (68.07)	47 (74.60)	36 (80.00)	
*Observed Data (n (%))*	256 (93.43)	153 (92.17)	60 (95.24)	43 (95.56)	0.693
*Missing Data (n (%))*	18 (6.57)	13 (7.83)	3 (4.76)	2 (4.44)	
***Household Characteristics***					
Household Asset Index (mean (SD))	0.01 (1.55)	0.01 (1.62)	-0.04 (1.37)	0.06 (1.56)	0.943
*Observed Data (n (%))*	270 (98.54)	164 (98.80)	62 (98.41)	44 (97.78)	0.795
*Missing Data (n (%))*	4 (1.46)	2 (1.20)	1 (1.59)	1 (2.22)	
Household Food Insecurity (n (%))					
Food Secure	85 (31.02)	46 (27.71)	23 (36.51)	16 (35.56)	0.236
Food Insecure	170 (62.04)	110 (66.27)	33 (52.38)	27 (60.00)	
*Observed Data (n (%))*	255 (93.07)	156 (93.98)	56 (88.89)	43 (95.56)	0.329
*Missing Data (n (%))*	19 (6.93)	10 (6.02)	7 (11.11)	2 (4.44)	
Ownership of Pig(s) (n (%))					
No	247 (90.15)	155 (93.37)	51 (80.95)	41 (91.11)	0.016
Yes	23 (8.39)	9 (5.42)	11 (17.46)	3 (6.67)	
*Observed Data (n (%))*	270 (98.54)	164 (98.80)	62 (98.41)	44 (97.78)	0.795
*Missing Data (n (%))*	4 (1.46)	2 (1.20)	1 (1.59)	1 (2.22)	
Ownership of Dog(s) (n (%))					
No	123 (44.89)	82 (49.40)	26 (41.27)	15 (33.33)	0.143
Yes	147 (53.65)	82 (49.40)	36 (57.14)	29 (64.44)	
*Observed Data (n (%))*	270 (98.54)	164 (98.80)	62 (98.41)	44 (97.78)	0.795
*Missing Data (n (%))*	4 (1.46)	2 (1.20)	1 (1.59)	1 (2.22)	
Tribe (n (%))					
Gonja	102 (37.23)	67 (40.36)	23 (36.51)	12 (26.67)	0.114
Konkomba	87 (31.75)	*44 (26.51)*	22 (34.92)	21 (46.67)	
Others	50 (18.25)	34 (20.48)	9 (14.29)	7 (15.56)	
*Observed Data (n (%))*	239 (87.23)	145 (87.35)	54 (85.71)	40 (88.89)	0.903
*Missing Data (n (%))*	35 (12.77)	21 (12.65)	9 (14.29)	5 (11.11)	
***Parent Characteristics (collected at 24 months)***					
Maternal Occupation (n (%))					
Farmer	130 (47.45)	65 (39.16)	34 (53.97)	31 (68.89)	0.108
Non-Farmer	52 (18.98)	34 (20.48)	12 (19.05)	6 (13.33)	
*Observed Data (n (%))*	182 (66.42)	99 (59.64)	46 (73.02)	37 (82.22)	0.007
*Missing Data (n (%))*	92 (33.58)	67 (40.36)	17 (26.98)	8 (17.78)	
Paternal Occupation (n (%))					
Farmer	142 (51.82)	74 (44.58)	33 (52.38)	35 (77.78)	0.845
Non-Farmer	18 (6.57)	10 (6.02)	5 (7.94)	3 (6.67)	
*Observed Data (n (%))*	160 (58.39)	84 (50.60)	38 (60.32)	38 (84.44)	<0.001
*Missing Data (n (%))*	114 (41.61)	82 (49.40)	25 (39.68)	7 (15.56)	
Maternal Education (n (%))					
No Formal Education	151 (55.11)	80 (48.19)	38 (60.32)	33 (73.33)	0.671
Primary	34 (12.41)	19 (11.45)	10 (15.87)	5 (11.11)	
*Observed Data (n (%))*	185 (67.52)	99 (59.64)	48 (76.19)	38 (84.44)	0.001
*Missing Data (n (%))*	89 (32.48)	67 (40.36)	15 (23.81)	7 (15.56)	
Paternal Education (n (%))					
No Formal Education	116 (42.34)	58 (34.94)	28 (44.44)	30 (66.67)	0.240
Primary	59 (21.53)	36 (21.69)	14 (22.22)	9 (20.00)	
*Observed Data (n (%))*	175 (63.87)	94 (56.63)	42 (66.67)	39 (86.67)	0.001
*Missing Data (n (%))*	99 (36.13)	72 (43.37)	21 (33.33)	6 (13.33)	
Ownership of Agricultural Land (n (%))					
No	16 (5.84)	13 (7.83)	3 (4.76)	0 (0.00)	0.128
Yes	251 (91.61)	149 (89.76)	58 (92.06)	44 (97.78)	
*Observed Data (n (%))*	267 (97.45)	162 (97.59)	61 (96.83)	44 (97.78)	0.868
*Missing Data (n (%))*	7 (2.55)	4 (2.41)	2 (3.17)	1 (2.22)	
***Environmental Characteristics***					
Location of Primary Water Source (n (%))					
In Household	15 (5.47)	11 (6.63)	2 (3.17)	2 (4.44)	0.852
Less than 50 metres away	139 (50.73)	82 (49.40)	32 (50.79)	25 (55.56)	
More than 50 metres away	116 (42.34)	71 (42.77)	28 (44.44)	17 (37.78)	
*Observed Data (n (%))*	270 (98.54)	164 (98.80)	62 (98.41)	44 (97.78)	0.795
*Missing Data (n (%))*	4 (1.46)	2 (1.20)	1 (1.59)	1 (2.22)	
Type of Sanitation Facility (n (%))					
Pit/Latrine	80 (29.20)	53 (31.93)	16 (25.40)	11 (24.44)	0.513
Bush/Field	190 (69.34)	111 (66.87)	46 (73.02)	33 (73.33)	
*Observed Data (n (%))*	270 (98.54)	164 (98.80)	62 (98.41)	44 (97.78)	0.795
*Missing Data (n (%))*	4 (1.46)	2 (1.20)	1 (1.59)	1 (2.22)	
Noticeable Garbage in Household (n (%))					
No	133 (48.54)	77 (46.39)	33 (52.38)	23 (51.11)	0.658
Yes	137 (50.00)	87 (52.41)	29 (46.03)	21 (46.67)	
*Observed Data (n (%))*	270 (98.54)	164 (98.80)	62 (98.41)	44 (97.78)	0.795
*Missing Data (n (%))*	4 (1.46)	2 (1.20)	1 (1.59)	1 (2.22)	
Study Communities (n (%))					
Atta Akuraa	41 (14.96)	26 (15.66)	9 (14.29)	6 (13.33)	0.051
Chiranda	13 (4.74)	11 (6.63)	1 (1.59)	1 (2.22)	
Gulumpe	86 (31.39)	51 (30.72)	21 (33.33)	14 (31.11)	
Jato Akuraa	16 (5.84)	7 (4.22)	6 (9.52)	3 (6.67)	
Kadelso	56 (20.44)	44 (26.51)	7 (11.11)	5 (11.11)	
Kawampe	49 (17.88)	22 (13.25)	14 (22.22)	13 (28.89)	
Mahama Akuraa	5 (1.82)	1 (0.60)	2 (3.17)	2 (4.44)	
Tahiru Akuraa	8 (2.92)	4 (2.41)	3 (4.76)	1 (2.22)	

#### Risk of future infections.

Future infections analysis focused on the 196 participants who presented for hookworm testing at all five time-points (Table 1b). Eighty-seven of these participants tested positive for hookworm infections on at least one occasion while the remaining 109 tested negative throughout. An initial assessment indicated that infections at the second and subsequent time-points were consistently higher among participants who were previously infected. Therefore, our models for the risk of future infections were formulated under the following assumptions: the probability of hookworm infection for any given child at the second and subsequent time-points may have a direct dependence on the previously observed hookworm infection, as well as other risk factors; and repeated transitions of hookworm infections for any given child could be treated as independent events (once the model is specified to adjust for previously observed hookworm infections) [64,65]. Data on household exposure variables were collected at baseline and eighteen months, hence to eliminate the effect of decay across time, we focused the analysis on modeling the risk of hookworm infections at 6 and 24 months. Using hookworm infection status at time-point *t*_*j*_ as our study outcome, we specified *Bernoulli* distributed first-order Markov transitional models adjusted for hookworm infections at time-point *t*_*j-1*_, as well as previously measured predictor variables at time-point *t*_*j-1*._

Our optimal set of predictor variables was identified by forward selection. Non-linearity in the continuous-value covariates was assessed with non-parametric smooth curves [66]. The risk of future hookworm infections was found to be non-constant across the pointwise values for height-for-age z-scores, blood hemoglobin levels and probability of nutrient adequacy. Hence, piecewise linear segments were defined for these covariates using estimated threshold values of -1.33 (SE: 0.53), 14.03 (SE:1.19) and 0.23 (SE:0.42), respectively (see supplemental materials). As five percent of the data on our optimal set of predictor variables was missing, we performed ten multiple imputations, and 50 iterations. Our multiple regression analyses involved ten complete data analyses on the imputed datasets and pooling of the results. Stepwise selection was employed in identifying the relevant predictors for each imputed model; the optimal model was adjusted for predictor variables selected at least fifty percent of the time [67,68].

#### Response to albendazole.

Study participants who tested positive for hookworm infections were assessed for their response to single dose (400mg) albendazole at four of the five data collection points. Of the 108 participants who tested positive for hookworm infection on at least one occasion, albendazole treatment was administered to 107 of them. A fourteen-day post-treatment re-assessment of infections was successfully carried out on at least one occasion for 83 participants, hence these participants became the focus of the analysis. There were 116 observations (one observation each for 60 participants and multiple observations for the remaining 23). We performed the analysis on a subset consisting of all 60 participants with single observations and the first observation for each of the 23 with multiple measures (see S1 Table).

Twenty (i.e., 24%) of the 83 participants had received albendazole treatments at a previous time-point for which post-treatment data were missing. We therefore created a dummy variable to account for the varying exposure levels associated with previous treatment events. Using albendazole treatment outcome as the response variable, probability of any given child experiencing an effective treatment was modelled in a discrete-time binary *complementary log-log* model with the number of received albendazole treatments as an offset [69].

A classification tree was employed in identifying the relevant set of predictors for treatment efficacy. A non-constant hazard was observed across the age range, with effective treatment being more likely in older children. Hence, piecewise linear segments for age were defined using an estimated threshold value of 9.66 years (SE: 1.22). As eight percent of the data on the optimal set of predictor variables were missing (Table 2), we performed ten multiple imputations of the data, after establishing that the missingness mechanism could be accepted as random. Stepwise selection was employed in identifying the relevant set of predictors in the ten imputed models. Our final model for treatment efficacy was adjusted for predictors selected at least 50 percent of the time [67,69].

### Adherence to STROBE guidelines

We utilized the STROBE statement checklist to ensure all recommended items for cohort studies are reported [70].

## Results

### Hookworm prevalence and intensity over the 24-month longitudinal study period

**Fig 2:**
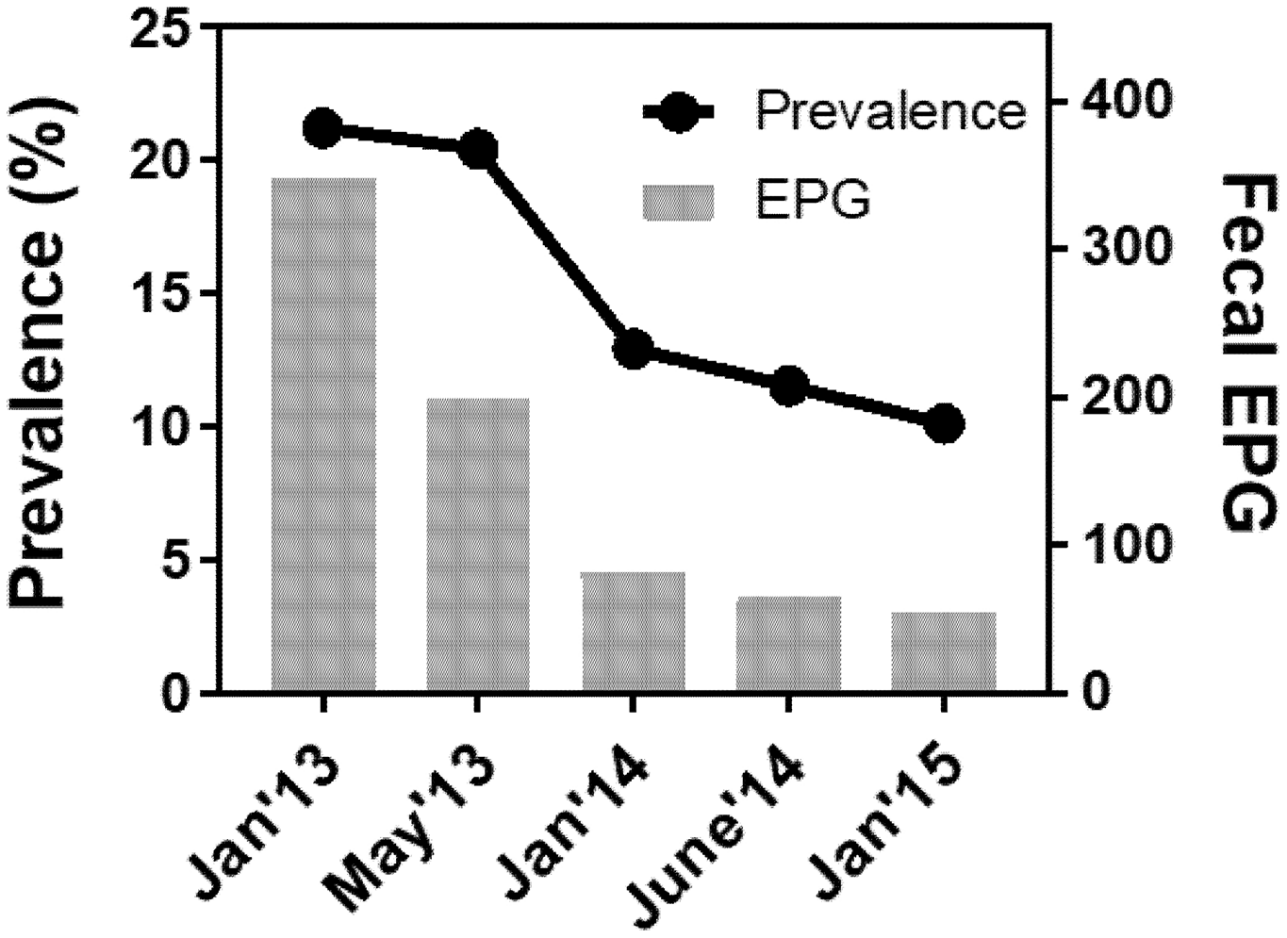
Prevalence (line) and intensity (bars) of hookworm infection. Prevalence and intensity (epg) were measured five times over the 24 month study period. Intensity (epg) is presented for children who were hookworm positive.

Prevalence and intensity of infections were highest at baseline, with a sharp decline in both from May 2013 to January 2014 (Fig 2). At baseline all participants had light infections except for one participant who had a moderate infection (2076 epg) and another who had a severe infection (6024 epg). At the remaining four time-points all children had < 1000 epg. Although the rates continued to decrease after January 2014, the decline was less pronounced, with only slight reductions observed in the subsequent measurements in June 2014 and January 2015. As the initial screening and treatment led to dramatic reductions in prevalence and intensity, the continued persistence raised questions about other factors within the community that may influence the reduction of hookworm infection over time.

### Risk factors for infection

Children from households with pigs, where household members tested positive for hookworm and where the household reported previous treatment for worms had higher rates of hookworm infection (Table 2). At the individual level, children who reported owning footwear had lower rates of hookworm infection, although reported frequency of wearing shoes was not associated with hookworm infection. There was significant heterogeneity in rates of infection across the eight communities, as between 20% and 85% of the participating children in any one community had negative fecal exams at all times.

### Future infections

#### Six months (Table 3a).

Infections at six months were 2.62 times higher among the baseline-infected children. Forty percent (19/47) of children who were infected at baseline were also infected at six months, while only 15.4% (23/149) of children who were not infected at baseline were newly infected at six months.

**Table 3:** (a) Predictors of Future Infection (0–6 months). (b) Predictors of Future Infection (18–24 months).

**(a)**						
**Model Terms**	**Bivariate Models**			**Final Model**		
	^**λ**^ **OR (95% CI)**	**SE**	**p-value**	**AOR (95% CI)**	**SE**	**p-value**
**Baseline Hookworm Infection**						
Non-Infected	1					
Infected	3.72 (1.78, 7.77)	0.37	0.000			
**Albendazole Treatment Outcome at Study Baseline**						
Non-Infected	1			1		
Treated Cured	1.83 (0.61, 4.89)	0.52	0.250	1.19 (0.31, 4.57)	0.680	0.795
Treated Non-Cured	10.96 (3.18, 43.92)	0.65	0.000	87.71 (9.20, 835.69)	1.142	0.000
Treated Unknown^a^	4.57 (1.23, 16.42)	0.65	0.019	8.93 (1.59, 50.10)	0.873	0.013
**Age**	1.13 (0.95, 1.34)	0.09	0.172	1.41 (1.08, 1.84)	0.135	0.011
***Height-For-Age z-scores <= -1.33*** ^**¥**^	0.60 (0.36, 1.03)	0.26	0.053			
***Height-For-Age z-scores > -1.33*** ^**¥**^	1.56 (1.07, 2.28)	0.19	0.020	2.93 (1.51, 5.68)	0.334	0.002
**Frequency of Wearing Footwear**						
Most Often	1			1		
Rarely	2.80 (1.18, 7.80)	1.03	0.030	3.13 (0.75, 1.31)	0.726	0.118
**Tribe**						
Gonja	1			1		
Konkomba	2.93 (1.27, 7.25)	0.44	0.015	2.44 (0.58, 10.35)	0.726	0.222
Others	2.16 (0.70, 6.49)	0.56	0.168	3.67 (0.61, 22.15)	0.905	0.154
***Probability of Nutrient Adequacy >0.23*** ^***¥***^	0.68 (0.47, 0.98)			0.17 (0.02, 1.69)	1.158	0.129
***Probability of Nutrient Adequacy ≤0.23***^***¥***^ ***(<0.23 relationship is linear, and OR approaches infinity)***	-^b^					
***Blood Haemoglobin Level <= 14.03g/dl*** ^***¥***^	1.32 (0.97,1.80)	0.16	0.081	1.90 (1.07, 3.37)	0.289	0.028
***Blood Haemoglobin Level > 14.03g/dl*** ^***¥***^	0.36 (0.03, 1.81)	0.95	0.277	0.06 (0.01, 0.78)	1.263	0.031
**Study Community**						
Atta Akura	1			1		
Chiranda	0.45 (0.02, 3.36)	1.17	0.497	0.85 (0.05, 14.26)	1.430	0.908
Gulumpe	0.79 (0.27, 2.50)	0.56	0.674	0.86 (0.17, 4.20)	0.806	0.847
Jato Akuraa	1.19 (0.21, 5.79)	0.82	0.835	0.60 (0.05, 6.60)	1.214	0.675
Kadelso	0.19 (0.03, 0.93)	0.87	0.057	0.14 (0.01, 1.78)	1.279	0.129
Kawampe	1.72 (0.56, 5.67)	0.58	0.353	2.92 (0.50, 17.13)	1.199	0.232
Mahama Akuraa	1.58 (0.07,19.65)	1.31	0.726	0.04 (0.00, 2.57)	2.152	0.127
Tahiru Akuraa	1.27 (0.15, 7.80)	0.96	0.805	5.53 (0.50, 60.93)	1.215	0.161
**Malaria Infection**						
Non-Infected	1			1		
Infected	0.46 (0.15, 1.17)	0.52	0.129	4.23 (0.99, 18.11)	0.734	0.052
**Gender**						
Female	1			1		
Male	1.95 (0.98, 3.99)	0.36	0.061	1.95 (0.75, 5.03)	0.480	0.167
**Household Treatment** ^**c**^						
Non-Treated	1					
Treated	0.46 (0.21, 0.95)	0.380	0.041			
**Asset Index**	0.88 (0.69, 1.11)	0.12	0.279			
**Noticeable Garbage in Household**						
No	1					
Yes	0.86 (0.43, 1.71)	0.35	0.664			
**Household Ownership of Dog(s)**						
No	1					
Yes	2.73 (1.31, 6.05)	0.39	0.010			
**Household Ownership of Pig(s)**						
No	1					
Yes	2.03 (0.67, 5.62)	0.53	0.186			
**Household Primary Sanitation Facility**						
Pit Latrine	1					
Bush/Field	1.09 (0.51, 2.45)	0.395	0.826			
**(b)**						
**Model Terms**	**Bivariate Models**			**Final Model**		
	**OR (95% CI)**	**SE**	**p-value**	**AOR (95% CI)**	**SE**	**p-value**
**Hookworm Infection at 18 Months**						
Non-Infected	1					
Infected	9.70 (3.53, 26.95)	0.514	0.000			
**Albendazole Treatment Outcome at 18 Months**						
Non-Infected	1			1		
Treated Cured	7.27 (2.20, 22.91)	0.589	0.001	6.36 (1.80, 22.44)	0.639	0.004
Treated Non-Cured	21.82 (3.30, 79.55)	0.965	0.001	33.51 (4.16, 270.06)	1.058	0.001
Treated Unknown	14.55 (0.55, 385.07)	1.448	0.064	35.50 (1.53, 824.74)	1.595	0.026
**Household Ownership of Dog(s) at 18 Months**						
No	1			1		
Yes	3.29 (1.22, 10.47)	0.538	0.027	5.05 (1.43, 17.85)	0.640	0.012
**BMI-For-Age at 18 Months**	1.99 (1.08, 3.87)	0.323	0.033	2.02 (0.99, 4.10)	0.359	0.052
**Household Treatment**						
Non-Treated	1					
Treated	1.09 (0.41, 3.10)	0.508	0.860			
**Height-For-Age (HAZ)**	1.63 (1.10, 2.44)	0.201	0.015			
**Probability of Iron Adequacy (PNA Iron)**	3.79 (1.14, 15.28)	0.649	0.040			
**Age**	0.97 (0.77, 1.21)	0.114	0.791			
**Gender**						
Female	1					
Male	1.80 (0.72, 4.75)	0.474	0.215			
**Tribe**						
Gonja	1					
Konkomba	2.78 (0.88, 10.56)	0.617	0.097			
Others	1.23 (0.16, 6.69)	0.895	0.816			
**Asset Index**	1.13 (0.77, 1.69)	0.202	0.538			
**Noticeable Garbage in Household**						
No	1					
Yes	1.31 (0.51, 3.48)	0.481	0.578			
**Household Ownership of Pig(s)**						
No	1					
Yes	2.31 (0.61, 7.19)	0.615	0.173			
**Household Primary Sanitation Facility**						
Pit Latrine	1					
Bush/Field	1.69 (0.53, 7.50)	0.652	0.420			
**Malaria Infection**						
Non-Infected	1					
Infected	1.31 (0.48, 4.18)	0.541	0.620			

^**λ**^**OR:** Odds Ratios.

^**§**^**AOR:** Adjusted Odds Ratios.

^**¥**^Defined Piecewise Linear Segments for Height-For-Age, Blood Haemoglobin Level and Probability of Nutrient Adequacy; each variable is continuous within the category.

^a^Consists of two collapsed levels: treated but lost to follow-up (n = 9); and infected but absent at the time of treatment (n = 2).

^b^The effect was flat.

^c^Household response that they had taken deworming medication in the previous year.

#### Bivariate effects at six months.

Hookworm infection at baseline more than tripled the odds of infection at six months (OR: 3.72; 95% CI:1.78, 7.77). Moreover, hookworm-infected children who received albendazole treatment at baseline were 3.53 times more likely to be infected six months later than non-infected children (OR: 3.53; 95% CI:1.68, 7.41). The odds of future infection varied with albendazole treatment outcome at baseline and ranged from 10.96 (95% CI:3.18, 43.92) for the non-cured children, 4.57 (95% CI:1.23, 16.42) for those that were lost to follow-up, and 1.83 (95% CI:0.61, 4.89) for those that were effectively cured of hookworm.

For every unit increase in baseline height-for-age z-scores above -1.33, the odds of future infection increased by 1.56 (95% CI: 1.07, 2.28). Likewise, every unit increase in baseline blood hemoglobin level up to 14.03g/dl was marginally associated with a 1.32 (95% CI: 0.97, 1.80) increased odds of infection at six months. Every unit increase in Probability of Nutrient Adequacy over 0.23 was associated with a 0.68 (95% CI: 0.47, 0.98) decreased odds of future infection at six months. Other statistically significant effects included household ownership of dog(s) (OR: 2.73; 95% CI: 1.31, 6.05), membership in the Konkomba tribe (OR: 2.93; 95% CI:1.27, 7.25), rarely wearing footwear (OR: 2.80; 95% CI: 1.18, 7.80), and residence in a household where member(s) had taken deworming medication in the previous year (OR: 0.46; 95% CI: 0.21, 0.95).

#### Final model: Future infection at six months.

Holding all other factors constant, the effect of albendazole treatment at baseline remained the dominant factor, with the highest odds occurring in children who still tested positive two weeks after receiving albendazole at baseline (OR: 87.71; 95% CI: 9.20, 835.69). The odds of future infection at six months also increased with age, HAZ > -1.33, blood hemoglobin level ≤ 14.03g/dl and malaria co-infection and decreased with blood hemoglobin levels > 14.03g/dl (Table 3a).

#### Twenty-four Months (Table 3b).

Forty-eight percent (10/25) of the observed infections at 24 months occurred in children who were infected at 18 months.

#### Bivariate effects at twenty-four months.

The odds of hookworm infection at 24 months in children who were also previously infected at 18 months was 9.70 (95% CI: 3.53, 26.95) times the odds for those who were uninfected at 18 months. Albendazole-treated children at 18 months were more likely to be infected at 24 months, with the odds varying from 7.27 (95% CI: 2.20, 22.91) for those who were effectively treated of infections, 21.82 (95% CI: 3.30, 79.55) for those that remained positive after treatment, and 14.55 (95% CI: 0.55, 385.07) for those that were lost to follow-up. A unit increase in height-for-age z-scores, BMI-for-age z-scores and PA Iron were linearly associated with increased odds of hookworm infection (HAZ: 1.63 (95% CI: 1.10, 2.44), BAZ: 1.99 (95% CI: 1.08, 3.87), and PA Iron: 3.79 (95% CI: 1.14, 15.28)). Participants from households that owned dog(s) at 18 months were 3.29 (95% CI: 1.22, 10.47) times as likely to be infected at 24 months compared to those from households without dog(s).

#### Final model (twenty-four months).

Adjusting for household ownership of dog(s) and BAZ, the odds of future hookworm infection at 24 months in children who received albendazole treatment at 18 months was 6.36 (95% CI: 1.80, 22.44) for those that were effectively treated of infections, 33.51 (95% CI: 4.16, 270.06) for those that experienced treatment failures, and 35.50 (95% CI: 1.53, 824.74) for those that were lost to follow-up (Table 3b).

### Response to albendazole

The assessment of albendazole treatment efficacy at 14 days post-treatment had an overall cure rate of 72.3% (60/83) (Table 4). Fecal egg reduction rate (ERR) in the 27.7% (23/83) of children who remained hookworm-positive after treatment ranged from 0–99%. Geometric average ERR did not vary significantly across time points.

**Table 4:** Predictors of Response to Single Dose Albendazole.

Model Terms	Bivariate Models			Final Model		
	*HR (95% CI)	SE	p-value	HR (95% CI)	SE	p-value
***Age (continuous) <=9.66 years*** ^***¥***^	1.14 (0.88, 1.47)	0.132	0.329			
***Age (continuous) >9.66 years*** ^***¥***^	1.40 (1.13, 1.72)	0.107	0.002	1.55 (1.18, 2.04)	0.134	0.002
**Household Food Insecurity**						
Food Secure	1			1		
Food Insecure	1.80 (0.94, 3.45)	0.331	0.074	2.26 (1.06, 4.81)	0.379	0.035
**Previous Albendazole Treatment (During the Study)**						
No	1			1		
Yes	0.34 (0.18, 0.65)	0.332	0.001	0.17 (0.07, 0.43)	0.460	0.000
**Time Since Last Meal (hours)**	1.01 (0.97, 1.05)	0.021	0.581	0.96 (0.91, 1.01)	0.026	0.130
**Height-For-Age**	0.87 (0.71, 1.06)	0.102	0.158			
**Asset Index**	1.10 (0.89, 1.36)	0.108	0.362			
**Household Treatment**						
Non-Treated	1					
Treated	0.87 (0.48, 1.58)	0.302	0.652			

*HR: Hazard Ratios. Offset: Number of albendazole treatment received during the study.

¥Defined Piecewise Linear Segments for Age; Age is continuous within the category.

#### Effect of individual factors.

Children aged over 9.66 years were found to be 40% more likely to experience an effective treatment (HR:1.40; 95% CI: 1.13,1.72). Children from food insecure households were 80% more likely to experience an effective treatment, as compared to those from the food secure households. This effect was of borderline significance (p = 0.07) in bivariate analysis. Treatment efficacy was 66% less likely at later time-points for children who had received multiple albendazole treatments over the course of the study, as compared to those who had not been treated previously during the study (HR: 0.34; 95% CI: 0.18, 0.65).

#### Final model (response to albendazole).

**Fig 3:**
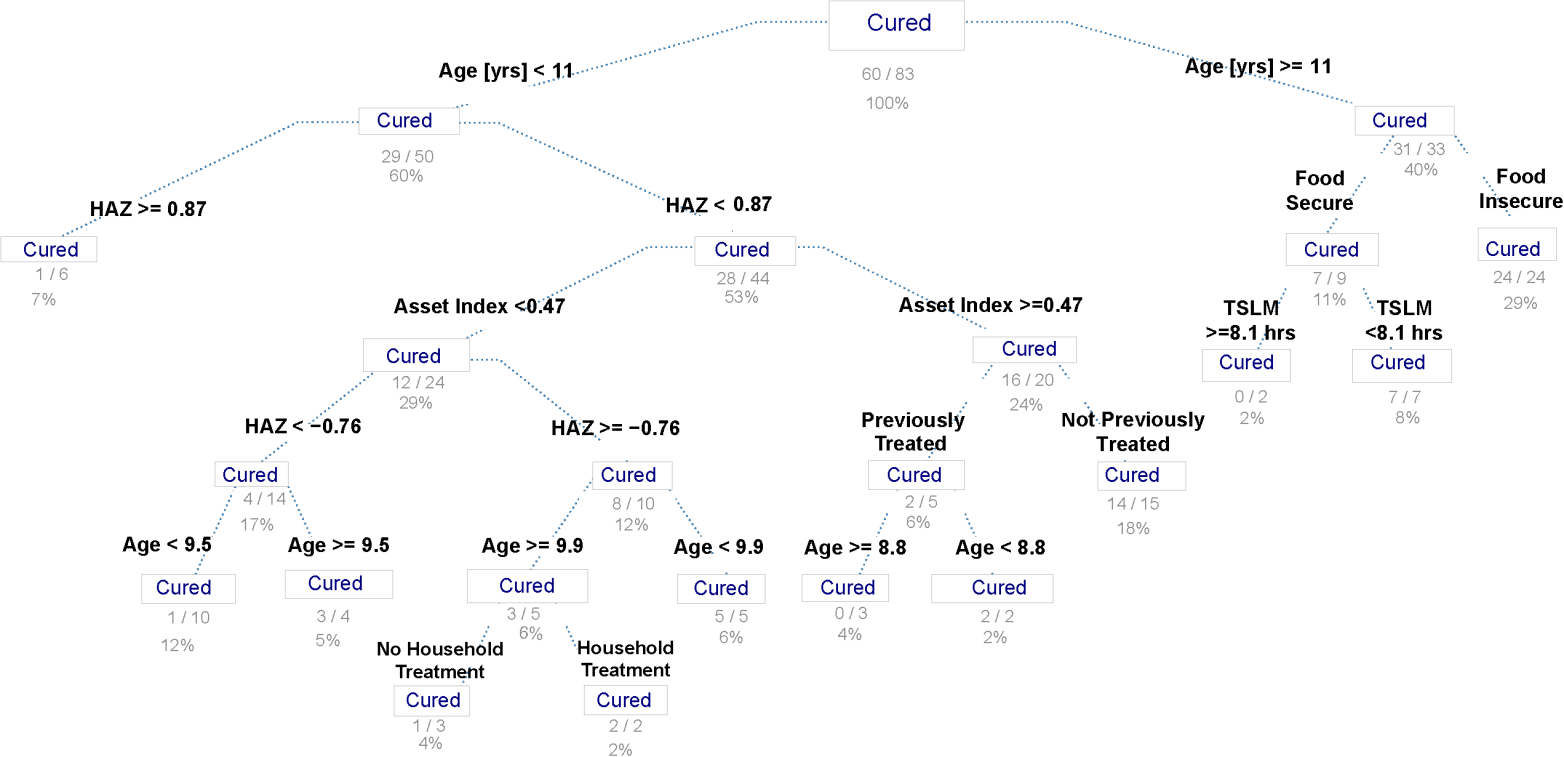
Partitioning Solution for Response to Single Dose Albendazole. The multiple occurrences of age and HAZ are indicative of non-linear effects between these variables and albendazole treatment outcome. Abbreviations: HAZ: Height-For-Age z-scores; TSLM: Elapsed time between albendazole treatment and last meal intake.

Holding all other factors constant, albendazole treatment efficacy was 55% more likely among children over 9.66y (HR:1.55; 95% CI: 1.18, 2.04). Children from households that were food insecure were 126% more likely to experience an effective treatment compared to those from the food secure households (HR: 2.26; 95% CI: 1.06, 4.81), and the partitioning analysis (Fig 3) suggests this effect was most influential in older children. Treatment efficacy was 83% less likely in children who received multiple treatments during the study (HR: 0.17; 95% CI: 0.07, 0.43). Our final model also included the elapsed time between albendazole treatment and last meal intake. While treatment efficacy increased non-significantly with increased time since last meal in bivariate analysis, after including food security, the direction of the relationship reversed. Treatment efficacy was 4% less likely with every hour increase in the elapsed time between albendazole treatment and last meal intake, i.e., an hour’s decrease in the elapsed time between albendazole treatment and last meal intake increased the probability of treatment efficacy by 4.2%. This effect was, however, statistically non-significant in the model (Table 4). The partitioning approach in Fig 3 identifies food security as influential for older children and time since last meal to be most influential among older children who also came from food secure households.

### Total number of infections

While 60.6% (n = 166) of participants (Table 1a), measured for an average of 4.36 (1.13) times (Table 2), were hookworm negative on every assessment, 2.6% (n = 7) tested positive four times and 1.1% (n = 3) were infected on all five tested occasions (Table 1b). The average number of observed times was significantly lower (4.36 (SD 1.13); p = 0.034) between children who were never infected and those that were infected on at least one tested occasion (4.63 (SD 0.88)).

#### Patterns of high frequency infections.

Of the three children infected at each of the five time points, only one had an instance where they did not receive the single dose albendazole. The remaining two received albendazole at each of the 5 timepoints. Moreover, two of the three children with infections at all time points tested hookworm negative two weeks after receiving single dose albendazole at least twice, highlighting the importance of exposure and reinfection in the overall hookworm burden. The seven children who tested positive four times each had one timepoint when they tested hookworm negative, and those occasions varied across the baseline, six-month, eighteen-month and twenty-four-month time points. In addition, each of these seven children tested hookworm negative at least once following receipt of single dose albendazole.

#### Final model: Influences on total numbers of infections.

Girls had a slightly lower risk of higher numbers of infections than boys, and children with a higher BAZ also had an increased risk of a higher number of total infections (Table 5). Tribe, particularly the Konkomba tribe, was associated with an increased risk of more infections, and households where member(s) reported taking deworming medication in the previous year had lower risk of higher total infections. The final model was adjusted for household assets and age of the child.

**Table 5:** Predictors of Total Number of Infections.

Model Terms	Final Model		
	*Adjusted IRR (95% CI)	SE	p-value
**Household Treatment**			
Non-Treated	1		
Treated	0.61 (0.36, 0.86)	0.128	0.018
**Tribe**			
Gonja	1		
Konkomba	1.85 (1.03, 2.68)	0.423	0.007
Others	1.06 (0.44, 1.68)	0.316	0.848
**BMI-For-Age**	1.31 (1.00, 1.61)	0.157	0.027
**Gender**			
Male	1		
Female	0.64 (0.39, 0.89)	0.127	0.024
**Age**	1.02 (0.92, 1.11)	0.048	0.754
**Household Asset Index**	0.90 (0.77, 1.03)	0.065	0.136

*IRR: Incidence Rate Ratios. Offset: Number of Measured Occasions.

Females had 36% fewer infections than males. We observed an increase of 31% in likelihood of an additional infection for every 1z-score increase in BAZ. Children from households belonging to the Konkomba tribe had 85% more infections when compared with those from Gonja tribe households. Holding all other factors constant, children from households where a household member reportedtaking deworming medication in the previous year had 39% fewer infections. For every additional year in age, a child had 2% more infections. For every unit increase in a household’s asset index, a child had 10% fewer infections.

## Discussion

While implementation of school-based deworming may have contributed to reduced rates of hookworm infection among schoolchildren in Africa [71], there are emerging concerns that MDA focused exclusively on school-aged children will not lead to elimination, and that additional strategies may be needed to achieve the WHO 2030 targets [24,72–74]. We report here on a two-year longitudinal study of schoolchildren. By focusing on school age children and following them through repeated cycles of assessment, treatment and re-exposure, we highlight additional patterns of risk that will be important for sustained control efforts and accomplishment of the WHO 2030 targets [74].

Of the 196 children who were screened at all five time points, 55% were negative throughout, with no clearly identifiable demographic factors associated with remaining uninfected. As we have not identified comparable reports of longitudinal monitoring of hookworm infection status in children, this is a critical finding, highlighting the importance of further work to explore potential exposure and susceptibility factors, including host risk behavior and genetics.

In assessing risk of total number of infections and future infections we hypothesized both environmental and nutritional factors as well as experience of previous treatment. We assessed environmental factors that are related to exposure, and infection and nutritional factors that relate to susceptibility to infection. There were significant environmental and nutritional predictors of total number of infections as well as infection at 6 and 24 months, although the specific variables were different.

Environmental influences included wearing shoes regularly, and a history of household members receiving deworming medications. Wearing of shoes has been associated with protection from infection, and the hypothesized pathway is generally through physical barriers to larval penetration of the foot and lower leg [75–79]. However, more rigorous evidence of causality is limited, with one attempted intervention showing limited impact, with extensive contamination between the control region and the intervention region, thus limiting the conclusions that could be drawn [80]. Receipt by other household members of deworming medications could indicate acknowledgement of worm infections in the household, although a recent sociology thesis in Ghana investigating self-medication practices in Ghanaian households in Accra found multiple households reported that everyone in the household took deworming medicine every three months as a precautionary measure [81].

We also report that dog ownership was positively correlated with hookworm infection among study participants. Previous studies have linked household dog or pig ownership with risk of infection and have identified dogs as potential vectors of hookworm, moving hookworm eggs from different locations into the household environment [7,45]. The association between dog ownership and STH infection highlights an important mechanism through which domesticated animals, lack of adequate sanitation, and chronically infected community members combine to promote ongoing transmission, highlighting the need for well integrated “OneHealth” approaches to disease control in endemic areas [82].

Nutritional influences included anthropometry, hemoglobin, and dietary intake. Children with higher HAZ or BAZ had increased odds of future infections, and children with higher BAZ had higher total numbers of infections. Interestingly, the relationship between HAZ and future infection at 6 months was nonlinear, and the AOR increased for children with HAZ > -1.3, which is in the low range of normal HAZ. Similarly, there was a nonlinear relationship with hgb, where hgb ≤ 14.03 was associated with an increased odds of future infection while hgb > 14.03 was associated with decreased odds of infection six months later. Previous cross-sectional studies of schoolchildren have found inconsistent relationships between anthropometry and hookworm infection, with some finding no association [83] and others finding higher prevalence of hookworm infection among malnourished children [84]. In the study reported here the effects of HAZ and BAZ were significant after controlling for age.

Anthropometric measures (i.e., HAZ and BAZ) represent a child’s unique combination of environment and genetics, and particularly the nutrition intake and infections the child has experienced [85,86]. Thus interpreting the finding that higher HAZ and BAZ are associated with increased risk of infection, is challenging and requires additional investigation. Poor dietary intake has generally been associated with increased risk of infections [7,9,83]. However, higher HAZ and BAZ in this study may mean healthier children who are more active, and thus at increased risk of exposure. Research differentiating the key drivers of anthropometric status in these participants could help clarify the relationship between anthropometry, nutritional status and infection risk.

This study also assessed dietary intake, and higher probability of adequacy of intake of nine micronutrients at baseline was associated with decreased risk of infection at six months, while higher probability of adequate iron at 18 months was associated with increased risk of infection at 24 months. With all markers of nutritional status we can expect nonlinear relationships with health outcomes, as nutrients can be deficient, adequate or in excess of the individual’s needs. Iron is particularly nonlinear, with deficiency compromising innate immune responses and excess leading to increased generation of reactive oxygen species and high levels of proinflammatory cytokines [87]. Hemoglobin, while flawed as a definitive marker of iron status, is often correlated with iron status [86]. Dietary iron and hemoglobin have been associated with susceptibility to infection in the hamster model, where both low and high iron intake were protective against hookworm infections, while animals receiving an intermediate-level iron diet were at highest risk of anemia [36]. In this study, children with a higher probability of consuming the WHO recommended intakes of iron at eighteen months were in the normal range of iron intake, and at increased risk of hookworm infection at 24 months.

General dietary adequacy, as assessed by the average probability of adequacy of micronutrients, assesses the nutrient intake profile of nine micronutrients (niacin, riboflavin, folate, thiamin, B6, vitamin C, iron, calcium and zinc). The PNA approach takes into consideration micronutrient needs for individuals that vary with age and gender, and expresses the probability that an individual’s nutrient needs are being met [55]. Decreased risk of hookworm infection at six months in bivariate analysis was associated with an increase in PNA9 for children with PNA9 > 0.23. In multivariate analysis PNA9 was selected to include in the model 80% of the time, although it was not significant in the final multivariate model. This suggests the variable includes some important explanatory power that is not captured in other variables, and that the final model included covariates that modified the effect of PNA9.

Previous infection and previous receipt of albendazole were important predictors of future infection. Given the variation in albendazole efficacy (i.e., overall cure rate of 72.3%) it is not possible to determine whether the association was due to reinfection or ongoing infections associated with sub-optimal drug response. Concerns have been raised about the potential for emerging drug resistance in human hookworms, due mostly to the more frequent resistance to benzimidazoles that has been observed in veterinary nematodes [88–92]. However, to date there is no clear evidence of genetically mediated benzimidazole resistance in human hookworms [93]. Analysis of single-nucleotide polymorphisms (SNPs) associated with benzimidazole resistance in *N. americanus* in samples from Kintampo North District have previously been published [44]. Although resistance-associated SNPs were present at higher frequency in post-treatment samples from individuals who were not cured, the presence of these mutations in pre-treatment samples did not predict the response to albendazole. More recent work in the Kpandai district in Ghana identified rare polymorphisms in the *N. americanus* isotype-1 β-tubulin gene from hookworm infected study subjects, but none were associated with treatment response [94]. These data demonstrate the need for additional work aimed at understanding the genetic basis of albendazole susceptibility and response to deworming in endemic populations, especially those exposed to repeated drug exposure.

Albendazole treatment efficacy was greater in older children and children from food insecure households. These relationships were nonlinear, with children over age 9.7y being the most responsive to albendazole. The partitioning analytical approach identified key risk factors sequentially, selecting the variable with the greatest explanatory power at each step. Thus the first step, by age, highlighted a greater effectiveness of albendazole in children over 9.7y. We would expect older children to have experienced more frequent hookworm infection and thus greater albendazole exposure than younger children, so multiple factors could underlie this observed relationship. Further research is needed to determine if there is an element of acquired immunity necessary to effectively expel hookworm from the body following treatment with single dose albendazole.

After partitioning by age, in children under 9.7y, the greatest explanatory power for understanding albendazole efficacy was a low household food security. While time since last meal was not a significant predictor, children from food insecure households were significantly more likely to have a longer time since the most recent meal, suggesting the possibility of a similar mechanism in terms of enhanced action of albendazole in the gut when less food is present [24,95]. Previously documented drug-nutrient interactions include impacts of meals (timing and ingredients) and individual nutritional status [95].

Malaria status at baseline was a significant predictor of infection at 6 months, and children with malaria had an OR of 4.2 (p = 0.42) risk of hookworm infection at six months after adjusting for age, treatment, HAZ, shoe wearing, PNA9, Hgb, community and gender. This increased risk of hookworm infection among children when infected with malaria has been shown previously in both cross-sectional and longitudinal studies, including among similar communities in Ghana [7,96–98]. At 24 months malaria infection was not a significant predictor of infection.

The results of this study point to the potential impact and limitations of targeted deworming strategies that have been advocated by the WHO and policy experts, and may still be relevant in Ghana and other hookworm endemic countries. The findings suggest that treatment of infected children more frequently than once per year may not achieve the necessary reductions in prevalence needed to interrupt transmission, which has been estimated at < 2% following treatment [99–101]. More recent studies than the one reported here suggest that STH infections persist in certain regions of Ghana [102,103], and that there is potential for infections to increase if at-risk communities, including school-age children, are no longer offered deworming treatment through existing government sponsored programs. It is worth noting that in 2026, according to its “Preventive Chemotherapy Data Portal” (https://www.who.int/data/preventive-chemotherapy), the WHO no longer recommends preventive chemotherapy for STH control in Ghana, presumably given estimates of national prevalence. Ultimately, therefore, it will be up to public health officials at the local, regional and national level to pursue the necessary surveillance and monitoring in order to identify measurable increases in STH transmission following cessation of annual mass drug administration. Given inconsistent access to adequate sanitation and hygiene infrastructure in many rural communities, the likelihood of such a “rebound” in Ghana may be substantial.

## Conclusions

This study reports on an innovative two-year longitudinal follow up of schoolchildren with repeated cycles of testing and treatment for hookworm infection. The study design allowed assessment of risk factors six months before assessment of infection status, strengthening the assessment of causal relationships between environmental, infection and nutritional risk factors. At both 6 and 24 months there were significant environmental and nutritional predictors of future infections, and there were also significant environmental and nutritional predictors of total number of infections, although the variables were different in each instance. Larger sample sizes and additional longitudinal studies are needed to strengthen understanding of the complex drivers of hookworm infections in children and communities.

### Limitations

This study had several limitations. As the total number of infections was small at 24 months (n = 21), the power to detect risk factors for future infections with smaller effect sizes was limited. In addition, we collected a large number of variables, and there was extensive missingness in key variables. We addressed the missingness by excluding variables with ≥30% missingness from the final analysis and by using a multiple imputation approach for other key variables with missing values.

### Strengths

This study has multiple strengths, including the analytical approach, two-year longitudinal assessment and treatment, extensive nutritional variables and rigorous data collection and monitoring. A number of continuous variables (i.e., HAZ, PNA, BAZ, Hgb) were nonlinear in their relationships with the outcomes of interest. To best utilize the information in these variables we chose to use multiple statistical approaches including generalized additive modeling and smooth plots, identifying segments of the continuous variables that could be utilized as separate linear functions. These approaches acknowledge the differential impacts of values of those variables in different regions of the range found in this population.

## Supporting information

S1 Fig Future Infections and Blood Haemoglobin. **S1a Fig:** Estimated non-parametric smoother of the relationship between blood haemoglobin level at baseline and the risk of hookworm infection at the six-month time-point (solid line). The 95% confidence bands are denoted by the two dotted lines. The dotted points represent the pointwise blood haemoglobin levels for hookworm negative (0) and hookworm positive (1) children. **S1b Fig:** Piecewise segments for blood haemoglobin level (solid line). The estimated threshold value for the breakpoint between the two segments (14.03) is denoted by the dotted vertical line. The dotted points represent the pointwise blood haemoglobin levels for hookworm negative (0) and hookworm positive (1) children.
(TIF)

S2 Fig Future Infections and Height-for-Age z scores. **S2a Fig:** The estimated smoothing curve of the relationship between Height-For-Age (HAZ) z-scores at baseline and the risk of hookworm infection at the six-month time-point (solid line). The two dotted lines denote the 95% confidence bands. The dotted points represent the pointwise HAZ z-scores for hookworm negative (0) and hookworm positive (1) children. **S2b Fig:** Piecewise segments for Height-For-Age z-scores (solid line). The dotted vertical line represents the estimated threshold value for the breakpoint between the two segments (i.e., -1.33). The dotted points represent the pointwise HAZ z-scores for hookworm negative (0) and hookworm positive (1) children.
(TIF)

S3 Fig Future Infections and Probability of Nutrient Adequacy. **S3a Fig:** Estimated smoother for the relationship between the average probability of nutrient adequacy, measured at baseline, and the risk of hookworm infection at the six-month time-point (solid line). The two dotted lines represent the 95% confidence bands. The dotted points represent the pointwise values of PNA9 for hookworm negative (0) and hookworm positive (1) children. **Supplemental Figure 3b:** Piecewise segments for the average probability of nutrient adequacy (solid line). The threshold value for breakpoint between the two segments (0.23) is denoted by the dotted vertical line. The dotted points represent the pointwise values of PNA9 average for hookworm negative (0) and hookworm positive (1) children.
(TIF)

S4 Fig Treatment Efficacy and Age. **S4a Fig:** Estimated smoother for the relationship between age and the probability of albendazole treatment efficacy (solid line) with the 95% confidence bands (dotted lines). The dotted points denote the pointwise ages of hookworm negative (0) and hookworm positive (1) children. **S4b Fig:** Piecewise segments for age (solid line). The threshold value for breakpoint between the two segments (9.66) is denoted by the dotted vertical line. The dotted points denote the pointwise ages of hookworm negative (0) and hookworm positive (1) children.
(TIF)

S1 Table Children were infected with hookworm and treated with single dose albendazole on different occasions and different numbers of times. (DOCX)
