# Supplementary material for: A 24 month longitudinal cohort study of hookworm infection among school-age children in Ghana: Predictors of persistent and repeated infection
Source: PLoS Negl Trop Dis. 2026 Jul 31;20(7):e0014492. doi: 10.1371/journal.pntd.0014492 (PMC13426945; doi:10.1371/journal.pntd.0014492)
Supplement: S1 Table — (DOCX) [file pntd.0014492.s005.docx]

| **Supplemental Table: Children were infected with hookworm and treated with single dose albendazole on different occasions and different numbers of times.** | | | | | |
| --- | --- | --- | --- | --- | --- |
| **Panel Size** | **N (%)** | **Average Number of Times Observed** | **Treatment Occasion (s)** | **Total Number:** | |
|  |  |  |  | **Children** | **Observations** |
|  |  |  |  |  |  |
| 1 | 60 (72.29) | 4.62 |  |  |  |
|  |  |  | T_1_ | 33 | 33 |
|  |  |  | T_3_ | 11 | 11 |
|  |  |  | T_4_ | 6 | 6 |
|  |  |  | T_5_ | 10 | 10 |
| 2 | 15 (18.07) | 4.93 |  |  |  |
|  |  |  | T_1_,T_3_ | 2 | 4 |
|  |  |  | T_1_,T_4_ | 3 | 6 |
|  |  |  | T_1_,T_5_ | 2 | 4 |
|  |  |  | T_3_,T_4_ | 5 | 10 |
|  |  |  | T_4_,T_5_ | 3 | 6 |
| 3 | 6 (7.23) | 4.83 |  |  |  |
|  |  |  | T_1_,T_3_,T_4_ | 2 | 6 |
|  |  |  | T_1_,T_4_,T_5_ | 1 | 3 |
|  |  |  | T_3_,T_4_,T_5_ | 3 | 9 |
| 4 | 2 (2.41) | 5 |  |  |  |
|  |  |  | T_1_,T_3_,T_4_,T_5_ | 2 | 8 |
|  |  |  |  |  |  |
| **TOTAL** | **83** |  |  | **83** | **116** |
| **T_1_**: Time-Point 1 (Baseline); **T_2_**: Time-Point 2 (Six Months Follow-up); **T_3_**: Time-Point 3 (12 Months Follow-up); | | | | | |
| **T_4_**: Time-Point 4 (18 Months Follow-up); **T_5_**: Time-Point 5 (24 Months Follow-up); | | | | | |
